# Patchy Distributions and Distinct Niche Partitioning of Mycoplankton Populations across a Nearshore to Open Ocean Gradient

**DOI:** 10.1128/Spectrum.01470-21

**Published:** 2021-12-15

**Authors:** Yingbo Duan, Ningdong Xie, Zhao Wang, Zackary I. Johnson, Dana E. Hunt, Guangyi Wang

**Affiliations:** a Center for Marine Environmental Ecology, School of Environmental Science and Engineering, Tianjin University, Tianjin, China; b Ocean College, Agricultural University of Hebei, Qinhuangdao, China; c Marine Laboratory, Duke University, Beaufort, North Carolina, USA; d Department of Biology, Duke University, Durham, North Carolina, USA; e Department of Civil and Environmental Engineering, Duke University, Durham, North Carolina, USA; f Key Laboratory of Systems Bioengineering (Ministry of Education), Tianjin University, Tianjin, China; University of Minnesota

**Keywords:** planktonic fungi, coastal waters, oceanographic gradients, diversity, community structure, spatial distribution

## Abstract

Evidence increasingly suggests planktonic fungi (or mycoplankton) play an important role in marine food webs and biogeochemical cycles. In order to better understand their ecological role and how oceanographic gradients from the coastal to open ocean shape the mycoplankton community, molecular approaches were used to study fungal dynamics along a repeatedly sampled, five-station transect beginning at the mouth of an estuary and continuing 87 km across the continental shelf to the oligotrophic waters at the boundary of the Sargasso Sea. Similar to patterns in chlorophyll *a*, fungal 18S rRNA gene abundance showed a sharp decrease from nearshore to offshore stations. While Shannon’s diversity was not statistically different across the transect, nonmetric multidimensional scaling (NMDS) ordination revealed that fungal communities at the nearshore station were significantly different from those at other stations. Even though spatial gradients were consistently strong, the shelf mycoplankton were more similar to those of the offshore communities when temperature was high (>20°C) and while they shifted toward the nearshore communities when temperature was low (<19°C), suggesting a role for additional seasonal factors (such as temperature) in shaping mycoplankton distributions. However, overall phylotype distributions were patchy with few taxa observed at all stations and the majority observed at a single station with the nearshore station exhibiting the largest number of exclusive phylotypes. Overall, our findings revealed the patchy spatial distributions and distinct niche partitioning of mycoplankton populations across a nearshore to open ocean gradient, which improved our understanding of fungal ecology in coastal waters.

**IMPORTANCE** Fungi are an important, but understudied, group of heterotrophic microbes in marine environments. Traditionally, fungi in the coastal ocean were largely assumed to be derived from terrestrial inputs. Yet here we find many fungal taxa are endemic to the open ocean environment but are rare or absent in nearshore waters, suggesting they are not washed into the ocean from the land. As observed for the bacterioplankton, coastal oceanographic gradients can function as habitat barriers to partition fungal communities. Compared to the bacterioplankton, however, the mycoplankton exhibit a much patchier distribution pattern, suggesting differential drivers and the potential for spatially/temporally limited habitats or strong density-dependent selection. Therefore, our results show that mycoplankton in the coastal ocean may play a significant but complementary role to that of the bacterioplankton.

## INTRODUCTION

Fungi can colonize a wide variety of environments, ranging from marine to freshwater and terrestrial habitats (1, 2). Their ecological success has been attributed to a diverse range of trophic strategies (3–5) and phenotypes (2, 6), which allow them to rapidly respond to changing substrate conditions (7) and to persist in adverse environmental conditions (8–11). In spite of their key role in nutrient cycling (12), their ecological functions, especially in marine environments, remain poorly characterized (3, 13).

As an integral component of marine microbiomes (14–16), mycoplankton are diverse and abundant in high-productivity and nutrient-rich coastal waters (15, 17–24). Ecologically, they are often assumed to be decomposers of terrestrial detritus (e.g., cellulose) and/or phytoplankton-derived organic matter with a notable contribution to secondary production in coastal marine ecosystems (15, 18). The highest fungal diversity has been observed in surface coastal waters rather than in the open ocean or deeper samples (18); and populations seem to be regulated by primary production, nutrients, and temperature (16, 21, 24). However, as many studies in coastal environments exhibit strong salinity gradients or terrestrial/riverine inputs, it has been difficult to differentiate fungal preferences for nearshore environments from passive introduction from terrestrial or freshwater sources (20, 21, 24). Complicating this interpretation, unlike most bacterioplankton, terrestrial fungi can survive large salinity changes (24, 25) and contribute to biogeochemical cycling in aquatic habitats (3, 26–28), as their chitin-rich cell walls are capable of overcoming the osmotic shock of a transition from fresh to seawater salinity (8). Our recent studies in the coastal regions of the Bohai Sea and the Pearl River Delta of the South China Sea suggest that the diversity and abundance of fungi are closely related to nutrient levels in coastal waters (19, 20). However, little is known about the distribution of fungi across oceanographic gradients from the coast to open ocean waters, specifically the contribution of terrestrial and nearshore fungal populations to mycoplankton in the open ocean.

**FIG 1:**
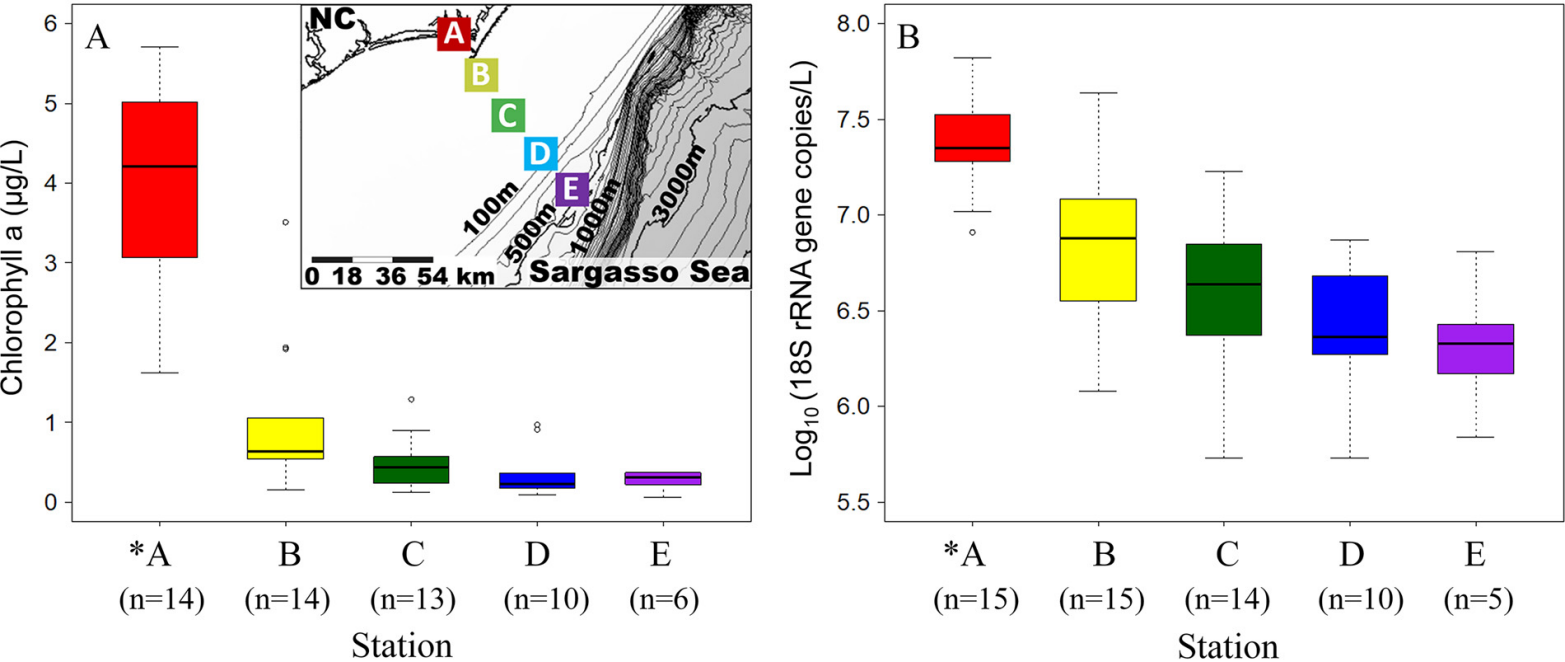
Spatial patterns across the Piver’s Island Coastal Observatory-Longitudinal Oceanographic Variability Experiment (PICO-LOVE) transects (July 2014-April 2016). (A) Box and whisker plot of chlorophyll *a* values and map (inset) to show spatial variability across strong coastal gradients from Station A at the mouth of the estuary out to Station E at the continental shelf break. (B) Box and whiskers plots of fungal abundance estimated by using fungi-specific 18S rRNA gene qPCR. For both panels, the box represents the 25th and 75th percentiles of its temporal variation; the horizontal line within the box represents the median value; the whiskers represent the lowest and highest values (excluding the outliers, labeled as open circles). In the parenthesis, n equals the number of samples collected at each station. The asterisk indicates that chlorophyll *a* and fungal 18S rRNA gene copy number at Station A was statistically different from the other four stations (ANOVA, *P* < 0.01; Tukey B, *P* < 0.01).

To that end, this study aims to understand the community structure of planktonic fungi across transects of the Piver’s Island Coastal Observatory-Longitudinal Oceanographic Variability Experiment (PICO-LOVE; Fig. 1, inset). Where the terrestrial influence is greatest, the nearshore station of the PICO-LOVE transect (Station A) is at the mouth of the Newport River Estuary, which is part of the Albemarle-Pamlico estuarine system, the second largest in the USA (29). The farthest offshore station is ∼87 km across the continental shelf adjacent to the oligotrophic waters of the Sargasso Sea, where environmental conditions are more stable and consistent with typical subtropical open ocean conditions. In this study, we applied high-throughput sequencing and quantitative PCR (qPCR) analyses as the primary tools to investigate fungal communities to understand how fungal populations are shaped by the nearshore to offshore ocean environmental gradients.

## RESULTS AND DISCUSSION

### Fungal abundance.

Here, in our investigation of the abundance and distribution of fungal communities across the PICO-LOVE coastal ocean transects, we maintained consistency with a previous study (29) by using the naming convention for station groupings: nearshore (Station A), shelf (Stations B and C), and offshore (Stations D and E). Fungal abundance estimated using fungi-specific 18S rRNA gene qPCR ranged from 5.38 × 10^5^ to 6.63 × 10^7^ copies L^−1^ and showed a decreasing trend from nearshore to offshore stations (Fig. 1). Fungal abundance at the nearshore Station A was significantly higher than at the other four stations (ANOVA, *P* < 0.01; Tukey B, *P* < 0.01) and this sharp decline from the coastline is consistent with higher chlorophyll *a* (Fig. 1A) and bacterial concentrations at Station A compared to the other stations (29). The fungal abundance at Station A was also more temporally dynamic, as observed for environmental parameters (e.g., temperature, chlorophyll *a*, and salinity) (29). Changes in fungal abundance were significantly correlated with multiple environmental variables (chlorophyll *a*, dissolved inorganic carbon, salinity, oxygen saturation, turbidity, and prokaryotic and picophotoeukaryotic abundance) (Table S1). A previous study of 3 years of weekly sampling at Station A revealed significant correlations between fungal abundance and salinity, chlorophyll *a,* and oxygen saturation (21); the data here reinforces the potential importance of these variables for fungal abundance across the coastal ocean.

### Fungal diversity.

**FIG 2:**
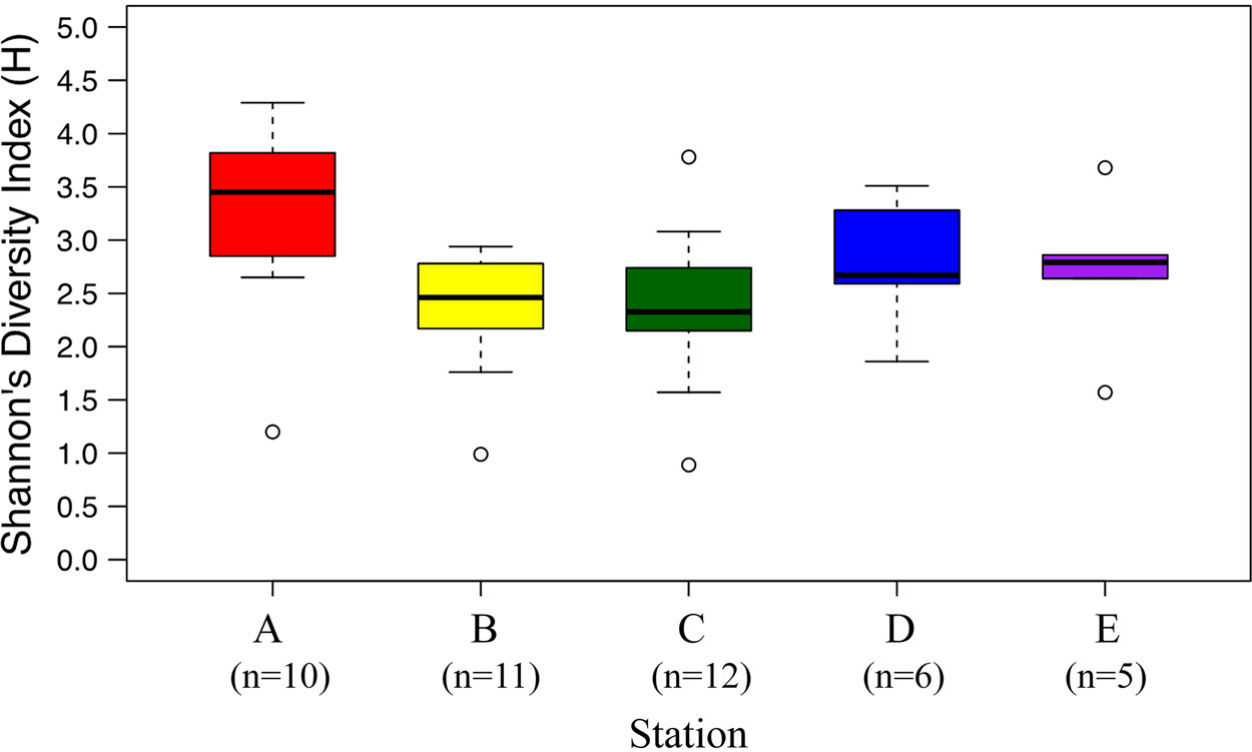
Shannon’s diversity using fungal internal transcribed spacer (ITS) 96% identity OTUs analysis of environmental DNA samples from Piver’s Island Coastal Observatory-Longitudinal Oceanographic Variability Experiment (PICO-LOVE) transect stations (July 2014-April 2016), with Station A closest to shore out to Station E at the continental shelf break. The box for each sampling site represents the 25th and 75th percentiles of observations; the horizontal line within the box represents the median value; the whiskers represent the lowest and highest values (excluding the outliers, labeled as open circles). In the parenthesis, n equals the number of samples collected at each station. Shannon’s diversity was not statistically different among the stations (ANOVA, *P* > 0.05).

We further investigated fungal community composition by sequencing the fungal internal transcribed spacer (ITS) region that was amplified using the PCR primers ITS1-F and ITS4. For the fungi, both Shannon’s diversity (Fig. 2) and OTU richness (Fig. S1), assessed using the entire data set of the cultured, uncultured known fungi and the predicted fungi, revealed that in spite of greater fungal 18S rRNA gene copy abundance and environmental variability closer to shore, diversity was not statistically different across the transect (ANOVA, *P* > 0.05) (Fig. 1 and 2). This is contrast to the previous studies that observed higher fungal diversity (richness of DGGE bands) closer to shore in the surface waters of a coastal upwelling ecosystem off central Chile (30) and at the Hawaiian coast (18), potentially due to different study regions or the low taxonomic resolution of the DGGE-based methods. The cultured fungal communities across the PICO-LOVE coastal ocean transects were composed of Ascomycota (61.76%), Basidiomycota (13.92%), Chytridiomycota (4.83%), Glomeromycota (0.68%), Mucoromycota (0.36%), Cryptomycota (0.04%), Kickxellomycotina (0.04%), Blastocladiomycota (0.01%), and the unclassified at phylum level (18.37%). While fungal diversity was significantly correlated with dissolved inorganic carbon and salinity in both PICO-LOVE (Table S1) and PICO time series studies (21), these variables are unlikely to directly influence fungal communities; for example, salinity was most variable at the nearshore site (ranging from 29.5 to 37.6) and therefore may serve as a proxy for either location or terrestrial influence (24).

### Fungal community partitioning across coastal gradients.

**FIG 3:**
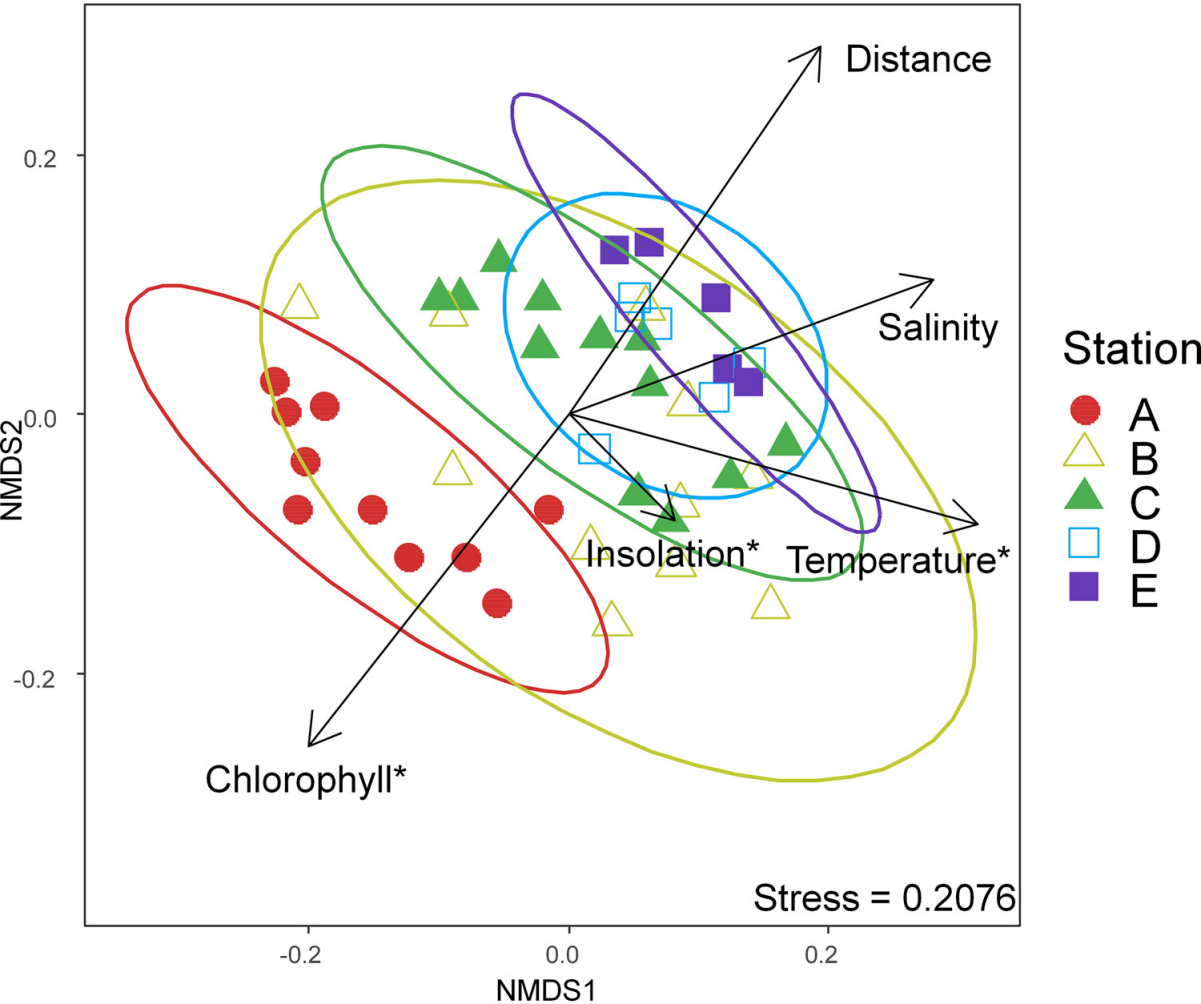
Nonmetric multidimensional scaling (NMDS) ordination based on Bray Curtis dissimilarity for fungal internal transcribed space (ITS) 96% identity OTUs in environmental samples from the Piver’s Island Coastal Observatory-Longitudinal Oceanographic Variability Experiment (PICO-LOVE) transect stations (July 2014-April 2016), with Station A closest to shore out to Station E at the continental shelf break. Each point corresponds to a single sample, with stations indicated by distinct shapes and colors. Ellipses show the 95% confidence intervals for the mean of each station. Vectors represent significant environmental factors in terms of marginal effects while those with asterisks are significant in terms of conditional effects (permutation tests in constrained ordination; *P* < 0.01).

Non-metric multidimensional scaling (NMDS) ordination revealed that fungal communities at the nearshore Station A were significantly different from those at other stations (PERMANOVA, *P* < 0.05; Fig. 3), but unlike the bacterioplankton, did not exhibit distinct shelf (Stations B and C) and offshore (Stations D and E) communities (29). Canonical-correlation analysis (CCA) revealed the main environmental factors associated with the community partitioning across nearshore to offshore habitats (Table S2) were water temperature, insolation, and chlorophyll *a* based on their conditional effect (999 permutations, *P* < 0.01), suggesting their potential importance in regulating the fungal community composition. Additionally, salinity and distance from shore were also significant in terms of their marginal effect (999 permutations, *P* < 0.01), suggesting distribution of fungal populations along these weakly correlated environmental gradients. Our observations suggest a joint impact of temporal and spatial factors on fungal communities; temperature was identified as the environmental factor with the strongest relationship to mycoplankton community composition, consistent with our findings in the nearshore time series and in other coastal locations (16, 21, 24, 31). In this study, we also observe spatial habitat segregation across nearshore to offshore gradients (PERMANOVA, *P* < 0.05; CCA marginal effect, *P* < 0.01) (Fig. 3 and Table S2); however, distance from shore may serve as a proxy for a number of environmental gradients, including terrestrial/freshwater influence, nutrients, primary producer biomass/primary productivity, water column depth, etc., each of which may influence microbial populations in distinct ways and care should be taken in the interpretation of any individual spatially associated variable as the proximal driver of community change.

**FIG 4:**
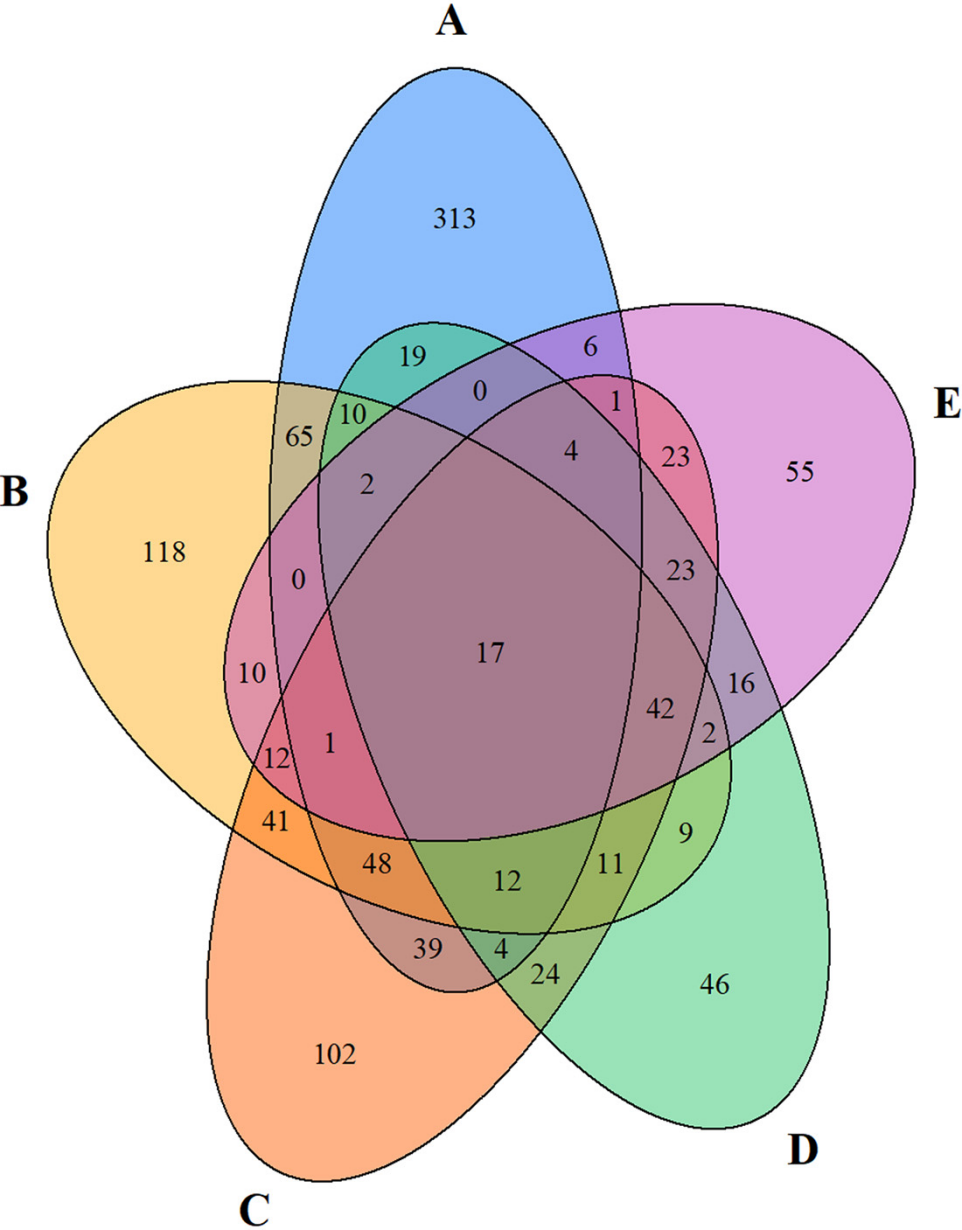
Venn diagram showing the spatial partitioning of fungal internal transcribed space (ITS) 96% identity OTUs in environmental samples from the Piver’s Island Coastal Observatory-Longitudinal Oceanographic Variability Experiment (PICO-LOVE) transect stations (July 2014-April 2016), with Station A closest to shore out to Station E at the continental shelf break.

In order to better understand how phylotypes are distributed across space and time, we further examined the spatial distribution of individual OTUs. Station A exhibited the largest number of exclusive phylotypes (Fig. 4), consistent with the nearshore site having the greatest temporal dynamics in environmental conditions and potential for wash-in of terrestrial and freshwater taxa. However, we also identified a number of shelf and/or offshore specific OTUs, suggesting these fungi are likely endemic to pelagic waters. Overall, there were few shared phylotypes across stations, i.e., only 1.58% of OTUs, representing 20.32% of total sequences, were observed at all stations (location-generalists) (Fig. 4), while the majority of OTUs were found only at one station (location-specialists). Potentially explaining some of this spatial segregation is that nearly 90% of the fungal OTUs, including those with high abundances, occurred in <10% of the libraries, suggesting patchy spatial and/or temporal distributions, consistent with previous observations in fungi (21) and fungus-like protists (32). The distribution pattern could be attributed to fungal preferences for large, patchily distributed particles (33). Most of location generalists (11 out of 17 OTUs) were cultured fungi (7 OTUs belonging to the phylum Ascomycota, 1 OTU to Basidiomycota, and other 3 OTUs unclassified at the phylum level) (Table S3). Within the phylum Ascomycota, most of location generalists were associated with the class Dothideomycetes, which were previously observed to be saprophytes, as well as parasites or symbionts of seagrasses or marine algae (34).

**FIG 5:**
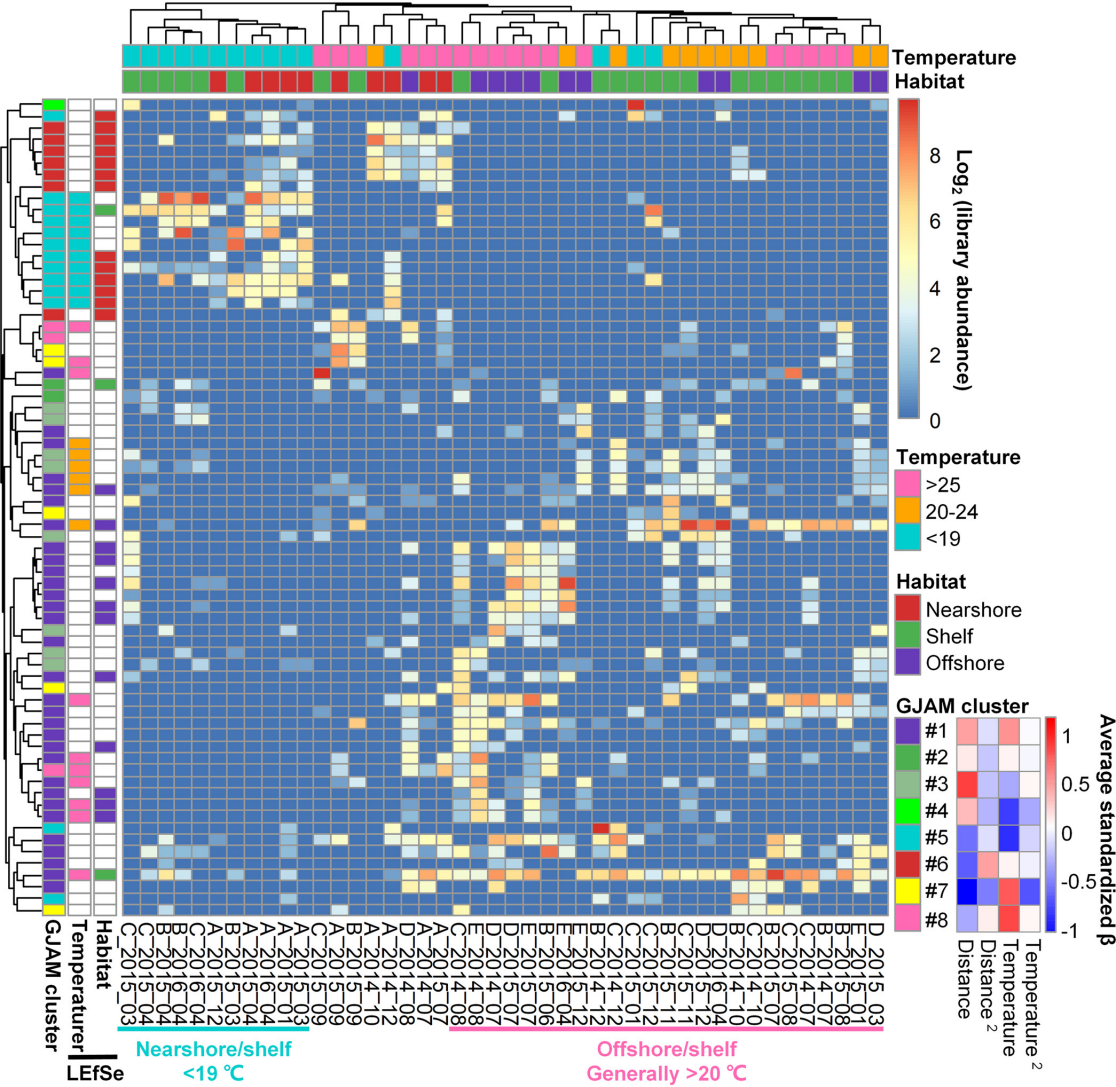
Library abundances and distribution patterns of the 70 fungal internal transcribed space (ITS) 96% identity OTUs, which occurred in ≥5 environmental samples and accounted for >0.1% of total sequences from the Piver’s Island Coastal Observatory-Longitudinal Oceanographic Variability Experiment (PICO-LOVE) transect stations (July 2014 -April 2016). The heatmap showed the log_2_(x + 1) transformed rarefied library abundances of these OTUs. Samples (*x* axis) and OTUs (*y* axis) were clustered by average correlations. Samples were labeled with water temperature ranges and habitat types (red: nearshore Station A; green: shelf Stations B and C; indigo: offshore Stations D and E). OTUs were labeled with GJAM clusters and LEfSe biomarkers. The GJAM standardized beta coefficients of temperature, distance, and their quadratics terms were averaged for members of each cluster and plotted in the figure legend.

In order to identify potential environmental preferences of fungal OTUs, we examined 70 prevalent OTUs (observed at least in 5/44 libraries with an average relative abundance > 0.1%), which represented 57.97% of the total sequences (Fig. 5). The heatmap of this subset of taxa indicated that the structure of fungal communities was mostly shaped by location; however, the shelf (Stations B and C) communities overlapped with either nearshore (Station A) or offshore (Stations D and E) communities (Fig. 5), reflecting the dynamic and transitional character of this environment. As observed in bacterioplankton from the same transects (29), the shelf (Stations B and C) mycoplankton were more similar to those of the offshore (Stations D and E) communities when temperature was high (>20°C), while they shifted toward the nearshore (Station A) communities when temperature was low (<19°C), suggesting that distributions are jointly shaped by temperature and factors associated with distance from shore. While we cannot definitely assign a cause, temperature appears to be a key variable for these microbes and the cooler nearshore and shelf environment is distinct from the always-warm offshore waters that are strongly influenced by the Gulf Stream. This key role for temperature is consistent with recent findings on mycoplankton in the Yellow Sea (35).

To gain greater insight into the distribution patterns of individual OTUs across these gradients in the coastal ocean, we used a common biomarker program which relies on *a priori* groupings (LEfSe) (36, 37) and a newer Bayesian modeling approach (GJAM) which jointly considers multiple environmental variables and does not require user-defined groups. These analyses focused on temperature and distance from shore, which both likely serve as proxies for complex suites of environmental factors. For example, temperature is correlated with both season and light levels, while distance from shore represents complex gradients in factors, including productivity, nutrients, terrestrial influence, and water column depth. The LEfSe analysis identified a number of biomarkers (OTUs) for nearshore, shelf, and offshore habitats and for different temperature ranges, suggesting distinct environmental preferences among fungal taxa in coastal waters (Fig. 5). The nearshore and offshore biomarkers (OTUs) included 13 and 12 members, respectively, suggesting that nearshore and offshore sites represent distinct habitats. Three “shelf-associated” OTUs were identified using LEfSe, but they were not strongly associated with Stations B and C. Previously, only a single algal OTU was associated with the shelf environment (29), suggesting that microbes generally do not exclusively associate with these spatially limited shelf habitats. In order to better understand the dynamics of fungal OTUs across spatiotemporal gradients, the generalized joint attribute modeling (GJAM) (38) was also used to group these 70 OTUs into 8 clusters based on their relationships with temperature and distance from shore. Cluster 1 (31 OTUs) was positively correlated with distance and temperature, suggesting a preference for offshore and warm conditions; and this group included all of the offshore biomarkers identified by LEfSe. Clusters also displayed different relationships with temperature, spanning positive (clusters 3, 4, and 5), insensitive (clusters 2 and 6), and negative (clusters 1, 7 and 8) relationships (Fig. 5). Similarly, members of clusters 5–8 were generally more abundant in nearshore samples (negative relationship with distance) but exhibited different temperature preferences. Clearly, these fungal OTUs have distinct relationships with environmental gradients, offering unique insights into which taxa are endemic to different aspects of the coastal ocean gradients.

**FIG 6:**
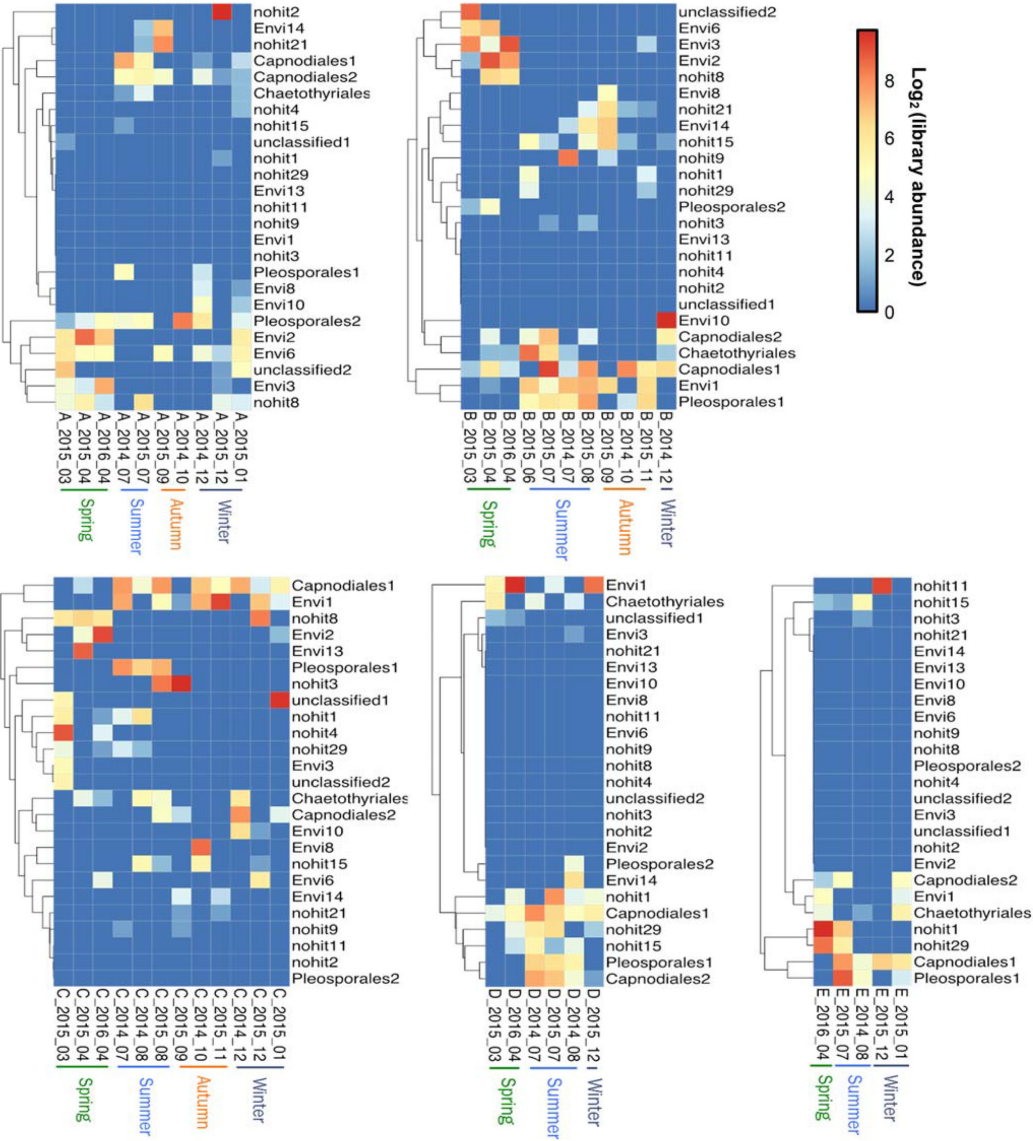
Spatial and temporal patterns of the 25 most abundant fungal internal transcribed space (ITS) 96% identity OTUs in environmental samples from the Piver’s Island Coastal Observatory-Longitudinal Oceanographic Variability Experiment (PICO-LOVE) transect stations (July 2014-April 2016), with Station A closest to shore out to Station E at the continental shelf break. The heatmap rows (OTUs) were labeled with the taxonomic assignments at order level if applicable. “Unclassified” represented the OTUs that matched sequences of cultured fungi without assignment at order level. “Envi” represented the OTUs that matched environmental sequences of uncultured fungi (with the closest taxonomy being fungi). “Nohit” represented the OTUs which did not match any sequences in the reference database and were considered “potential fungi.” The heatmap showed the log_2_(x + 1) transformed rarefied library abundances of these OTUs. Plots A-E correspond to Stations A-E, respectively.

To gain further insight into fungal temporal distributions in different stations, the 25 most abundant OTUs were examined to uncover variability within each station (Fig. 6). These OTUs represent 48.93% of total sequences in the data set, and the majority (18 of 25 OTUs) of them belonged to either the uncultured known fungi or predicted new fungi. These plots reveal likely seasonality at Stations A and B; but seasonal patterns were less obvious at Stations C, D, and E, where the environmental conditions were relatively stable.

## CONCLUSIONS

Historically, fungi in the coastal ocean were assumed to be largely washed in from soil; yet increasingly fungi are recognized to be important planktonic heterotrophs in marine environments (24). Here, we observed significant decline in total abundance but not diversity of the mycoplankton across a gradient spanning the nutrient-rich nearshore waters to the oligotrophic open ocean. Although fungal populations exhibit patchier spatial and temporal patterns compared to the bacterioplankton (29), we similarly identified fungal populations with distinct nearshore and offshore preferences. Particularly, many fungal taxa were found to be associated with the open-ocean environments but rare or absent in the nearshore waters, suggesting they are likely endemic to these regions. Compared with the bacterioplankton, the patchier distributions of heterotrophic eukaryotes (21, 32), will make it more difficult to predict how these taxa will respond to climate change. Future research directions could address whether this patchiness is largely due to resources (e.g., marine snow, algal blooms) or density-dependent selection that leads to rapid bloom declines. Whatever the origin, these generally ephemeral mycoplankton blooms suggest that their rapid response may complement the ecology of heterotrophic bacteria in the coastal ocean.

## MATERIALS AND METHODS

### Seawater sampling and environmental characterization.

PICO-LOVE (Piver’s Island Coastal Observatory-Longitudinal Oceanographic Variability Experiment) transects start at the mouth of the Newport River estuary adjacent to the Beaufort Inlet (Station A; PICO time series sampling station) (21) and proceed orthogonally across depth contours to the edge of the continental shelf break (Station E), spanning the nearshore Station A (34.7181°N 76.6707°W), shelf Stations B (34.6084°N 76.6708°W) and C (34.3584°N 76.4725°W), and offshore Stations D (34.1944°N 76.3328°W) and E (34.0345°N 76.1972°W), with stations ∼22 km apart and the most distant Station E 87 km from the coast (Fig. 1, inset).

Transect cruises were performed monthly or quarterly from July 2014 until April 2016, but due to field conditions, not all five stations were sampled for all transects (Table S5). Near surface water 1–4 L (1 m depth) was filtered through 0.22 μm Sterivex filters (Millipore) and the resulting filters were stored at −80°C until DNA extraction. Methods for determination of surface water temperature, turbidity, oxygen saturation, oxygen, insolation, pH, salinity, dissolved inorganic carbon, and chlorophyll *a* concurrent with seawater sampling were as described previously (39, 40).

### DNA extraction and fungal ITS amplicon sequencing.

The Gentra Puregene Yeast/Bacteria kit (Qiagen) coupled with bead beating for 60 s was used for genomic DNA extraction, followed by inhibitor cleaning with the Zymo OneStep PCR inhibitor removal kit and DNA quantification by a Nanodrop ND-100. The fungal internal transcribed spacer (ITS) region was amplified using the PCR primers ITS1-F 5′-CTTGGTCATTTAGAGGAAGTAA-3′ and ITS4 5′-TCCTCCGCTTATTGATATGC-3′ (41) with added bar codes and Illumina adapters. 25 μl PCRs contained 0.625 U of Jumpstart *Taq* (Sigma), 2.5 μM each primer, and ∼20 ng of DNA template. The PCR mixture was cycled at 94°C for 4 min, 30 cycles at 94°C for 30 s, 56°C for 40 s, and 72°C for 60 s, followed by a final extension of 5 min at 72°C. PCR products were verified by agarose gel electrophoresis. Thereafter, triplicate PCRs per sample were pooled and purified using the QIAquick PCR purification kit (Qiagen). The resulting PCR products were quantified using a Qubit (Invitrogen). Finally, 10 ng of each library was pooled and then purified using Agencourt AMPure XP beads (Beckman Coulter). MiSeq (Illumina) 2 × 250 bp sequencing was conducted at Duke’s Genome Sequencing and Analysis Core Facility.

### Processing of fungal ITS sequences.

The resulting sequences were demultiplexed and assigned to corresponding samples based on their barcode sequences using CASAVA software (Illumina), and the demultiplexed raw sequence data (without barcodes and primers) were deposited in NCBI as part of BioProject PRJNA437132. Further sequence analyses were conducted using USEARCH v8 (42). To maintain the highest sequence quality, only the forward read was used in this analysis (24). Sequences were further processed as follows: all the sequences were first truncated to 200 bp after which the reads quality dropped dramatically, and then the truncated sequences were trimmed at Phred quality (Q) of 30 using a 10-bp running window; a total quality score threshold was applied to filter reads with expected errors >1 or any reads that were trimmed to a length <200 bp during the quality filtering; singleton sequences were excluded from further analyses; the remaining sequences were assigned to OTUs at 98% pairwise identity using the centroid-based clustering UPARSE-OTU algorithm (43), with chimeras removed simultaneously using UCHIME (44); and OTUs occurring less than five times (i.e., sum of sequences across all libraries <5) in the data set were removed.

After quality filtering, a total of 1,292,785 sequences were obtained from the 56 fungal ITS gene libraries and classified into 2,537 OTUs. Taxonomy was assigned to OTUs using BLAST against the NT database (2 May 2018) using the top non-environmental hit, as described in the previous study (21). Of these OTUs, 819 (32.28%) had top matches with cultured fungi, 465 (18.33%) with uncultured fungi (but the closest taxonomy being fungi), 760 (29.96%) with ITS sequences from other eukaryotes, which were considered non-fungi OTUs and removed, and the rest 493 (19.43%) did not match any sequences in the database, which were considered potential fungal OTUs and retained for downstream analyses. We also applied a more conservative approach and only analyzed the sequences that definitively matched known fungi and the conclusions are broadly consistent; thus we include ITS sequences without matches to other eukaryotes as “potential fungi” in subsequent analyses; this likely overestimates the true fungi, but as these are fungal primers, we error on the side of including these potential new fungal groups rather than excluding poorly described diversity in this clade (45). It should also be aware that the analyses were done using an older database; it is likely that many new sequences have been added since then. The final OTU table was rarefied to 1,000 sequences per library for downstream analysis.

### Analyses of fungal diversity and community structure.

OTU richness was calculated using QIIME 1.9 (46). Shannon’s diversity index was computed using the *vegan* R package (47). The nonmetric multidimensional scaling (NMDS) ordination for the comparison of fungal community at each station was performed and visualized in R (47), fitted with significantly associated environmental variables in terms of marginal or conditional effects (999 permutations, *P* < 0.01) which were determined by canonical-correlation analysis (CCA) in Canoco 5 (48). In order to identify recurrent patterns in fungal communities, we extracted 70 prevalent fungal OTUs, which were observed at least in 5 (of 44 total) libraries and represented >0.1% of total sequences, and applied generalized joint attribute modeling (GJAM) to predict the relationships of these prevalent fungal OTUs with environmental factors (distance and temperature) using the GJAM v. 2.2.6 package in R (38). Iteration was set at 20,000 and burn in at 10,000, and both linear and quadratic terms were included in the model. We also applied linear discriminant analysis (LDA) effect size (LEfSe) (36, 37) to identify biomarkers for nearshore, shelf, and offshore habitats or temperature ranges, based on the normalized abundance data of the 70 prevalent OTUs. The one-against-all strategy was applied for the multiclass analysis, considering alpha values for the factorial Kruskal-Wallis test among classes <0.05 and logarithmic LDA scores >2 as significant (36, 37). The affiliations of each OTU to the GJAM clusters and LEfSe (biomarker) groups were visualized using the *pheatmap* R package, alongside a heatmap clustered based on community similarity to illustrate the distribution patterns of these prevalent fungi.

### Quantitative PCR of the fungal 18S rRNA gene.

Quantitative PCR (Q-PCR) assessed the total abundance of the fungal 18S rRNA genes. Primers FR1 (5′-AICCATTCAATCGGTAIT-3′) and FF390 (3′-CGATAACGAACGAGA CCT-5′) (24, 49) were used with the SYBR Premix *Ex Taq* (TaKaRa, Japan). The 10 μl reaction volume contained 1× SYBR Premix *Ex Taq*, 0.25 μM each primer, and ∼10 ng of DNA template. The Q-PCR was performed on a DNAEngine Peltier Thermal Cycler with a Chromo4 Real-Time PCR Detector (Bio-Rad, USA). The reactions were amplified with an initial denaturation at 95°C for 2 min, followed by 40 cycles of 95°C for 5s, annealing at 46°C for 30s and elongation at 72°C for 30s. Standard curves were constructed using known amounts of standard linearized plasmid, a combination of the pTOPO-TA vector (Gene-better, Beijing, China) and the target gene derived from genomic DNA of *Rhodosporidium diobovatum*.

### Data Availability.

Demultiplexed raw sequence data (without barcodes and primers) and corresponding environmental metadata were deposited in NCBI as part of BioProject PRJNA437132.
