# Supplementary material for: Patchy Distributions and Distinct Niche Partitioning of Mycoplankton Populations across a Nearshore to Open Ocean Gradient
Source: Microbiol Spectr. 2021 Dec 15;9(3):e01470-21. doi: 10.1128/Spectrum.01470-21 (PMC8672894; doi:10.1128/Spectrum.01470-21)

Supplementary Information

Patchy distributions and distinct niche partitioning of mycoplankton populations across a nearshore to open ocean gradient

Yingbo Duan[1,2#], Ningdong Xie[1,3#], Zhao Wang[3], Zackary I. Johnson[3,4], Dana E. Hunt[3,4], Guangyi Wang[1,5*]

[1]Center for Marine Environmental Ecology, School of Environmental Science and Engineering, Tianjin University, Tianjin 300072, China

[2]Ocean College, Agricultural University of Hebei, Qinhuangdao 066000, China

[3]Marine Laboratory, Duke University, Beaufort NC USA

[4]Biology and Civil & Environmental Engineering Departments, Duke University, Durham NC USA

[5]Key Laboratory of Systems Bioengineering (Ministry of Education), Tianjin University, Tianjin 300072, China

[#]Denotes equal authorship

Running title: Fungal communities across nearshore to open oceans

*Corresponding author:

Tel: (86) 022-8740210

Fax: (86) 0755-8740210

E-mail: gywang@tju.edu.cn

Table S1. The Pearson product-moment correlation coefficient (r) of environmental variables with the fungal 18S rRNA gene abundance and Shannon's diversity of fungal communities in PICO-LOVE transects. The numbers in bold indicate the significant correlations ($p < 0.05$).

**Environmental Variables**	**Abundance**		**Shannon's Diversity**	
	r	P	r	P
pH	-0.233	0.087	-0.258	0.108
Chlorophyll	0.651	**0.000**	0.183	0.233
Bacteria	0.545	**0.000**	0.365	**0.015**
Synechococcus	0.261	0.056	-0.111	0.501
Picocyanobacteria	0.014	0.919	-0.026	0.875
Picophotoeukaryotes	0.625	**0.000**	0.207	0.205
DIC	-0.456	**0.000**	-0.334	**0.035**
Insolation	-0.155	0.243	0.021	0.895
Salinity	-0.572	**0.000**	-0.314	**0.038**
Oxygen	0.099	0.475	-0.210	0.188
OxygenSaturation	-0.395	**0.003**	-0.157	0.326
Turbidity	0.641	**0.000**	0.317	**0.046**
Temperature	-0.200	0.129	0.012	0.940

Table S2. The canonical correspondence analysis (CCA) marginal and conditional term effects of environmental factors to the composition of all fungal OTUs.

Marginal Term Effects

Name	Explains %	pseudo-F	P	P(adj)[1]
**Temperature[2]**	**3.9**	**1.7**	**0.001**	**0.002**
**Salinity**	**3.4**	**1.5**	**0.003**	**0.004**
**Distance**	**3.3**	**1.4**	**0.001**	**0.002**
**Chlorophyll**	**3.3**	**1.4**	**0.001**	**0.002**
**Insolation**	**2.9**	**1.3**	**0.002**	**0.003**
Bacteria	2.5	1.1	0.196	0.196

Conditional Term Effects

Name	Explains %	pseudo-F	P	P(adj)[1]
**Temperature[2]**	**3.9**	**1.7**	**0.001**	**0.003**
**Insolation**	**3.0**	**1.3**	**0.001**	**0.003**
**Chlorophyll**	**3.0**	**1.3**	**0.008**	**0.016**
Salinity	2.7	1.2	0.143	0.172
Distance	2.6	1.2	0.096	0.144
Bacteria	2.0	0.9	0.731	0.731

[1] The significance was tested by 999 permutations and adjusted by false discovery rate.

[2] Variables in bold show significant effects ($P < 0.05$) after adjustment for multiple hypothesis testing.

Table S3. OTUs in Fig. 4 observed at all five stations of the PICO LOVE transects, with 11 cultured, 4 uncultured and 2 predicted fungi identified.

Universal OTUs	Fungi	Phylum	Class	Order	Family	Genus	
OTU_10	Cultured	Ascomycota	Dothideomycetes	Capnodiales	Cladosporiaceae	Cladosporium	Cladosporium cladosporioides
OTU_27		Ascomycota	Dothideomycetes	Capnodiales	Cladosporiaceae	Cladosporium	Cladosporium parahalotolerans
OTU_50		Ascomycota	Dothideomycetes	Capnodiales	Cladosporiaceae	Cladosporium	Cladosporium domesticum
OTU_124		Ascomycota	Dothideomycetes	Capnodiales	Teratosphaeriaceae		Devriesia sp. OUCMBI101247
OTU_79		Ascomycota	Dothideomycetes	Dothideales	Teratosphaeriaceae	Hortaea	Hortaea werneckii
OTU_22		Ascomycota	Dothideomycetes	Pleosporales	Pleosporaceae	Alternaria	Alternaria tenuissima
OTU_38		Ascomycota	Eurotiomycetes	Chaetothyriales	Trichomeriaceae		Knufia petricola
OTU_348		Basidiomycota	Malasseziomycetes	Malasseziales	Malasseziaceae	Malassezia	Malassezia restricta
OTU_1003		Unclassified					
OTU_250		Unclassified					
OTU_1293		Unclassified					
OTU_109	Uncultured	Basidiomycota	Agaricomycetes				
OTU_137		Basidiomycota	Agaricomycetes				
OTU_564		Basidiomycota	Agaricomycetes				
OTU_308		Uncultured fungi					
OTU_19	Prodicted	Prodicted fungi					
OTU_112		Prodicted fungi					

Table S4. The canonical correspondence analysis (CCA) marginal and conditional term effects of environmental factors to the composition of the 70 most prevalent fungal OTUs.

Marginal Term Effects

Name	Explains %	pseudo-F	P	P(adj)[1]
**Temperature[2]**	**6.9**	**3.1**	**0.001**	**0.002**
**Distance**	**5.1**	**2.2**	**0.001**	**0.002**
**Chlorophyll**	**5.0**	**2.2**	**0.001**	**0.002**
**Salinity**	**4.6**	**2.0**	**0.005**	**0.006**
**Insolation**	**3.9**	**1.7**	**0.002**	**0.003**
Bacteria	3.0	1.3	0.142	0.142

Conditional Term Effects

Name	Explains %	pseudo-F	P	P(adj)[1]
**Temperature[2]**	**6.9**	**3.1**	**0.001**	**0.002**
**Insolation**	**4.4**	**2.0**	**0.001**	**0.002**
**Distance**	**4.1**	**1.9**	**0.001**	**0.002**
**Chlorophyll**	**3.4**	**1.7**	**0.015**	**0.023**
Salinity	2.9	1.4	0.141	0.169
Bacteria	1.7	0.8	0.633	0.633

[1] The significance was tested by 999 permutations and adjusted by false discovery rate.

[2] Variables in bold show significant effects ($P < 0.05$) after adjustment for multiple hypothesis testing.

Table S5. The detailed information of samples including the specific cruise time and which samples were missing due to "field conditions".

Cruise	Year	Month	Day		Cruise	Year	Month	Day		Cruise	Year	Month	Day	
**605A**	2014	7	29		**637A**	2015	1	29		**672A**	2015	8	20	
**605B**	2014	7	29		**637B**	2015	1	29		**672B**	2015	8	20	
**605C**	2014	7	29		**637C**	2015	1	29		**672C**	2015	8	20	
**605D**	2014	7	29		**637D**	2015	1	29		**672D**	2015	8	20	
**605E**				missing	**637E**	2015	1	29		**672E**	2015	8	20	
**609A**	2014	8	15		**646A**	2015	3	25		**677A**	2015	9	17	
**609B**	2014	8	15		**646B**	2015	3	25		**677B**	2015	9	17	
**609C**	2014	8	15		**646C**	2015	3	25		**677C**	2015	9	17	
**609D**	2014	8	15		**646D**	2015	3	25		**677D**	2015	9	17	
**609E**	2014	8	15		**646E**				missing	**677E**				missing
**615A**	2014	9	18		**651A**	2015	4	24		**684A**	2015	11	1	
**615B**	2014	9	18		**651B**	2015	4	24		**684B**	2015	11	1	
**615C**				missing	**651C**	2015	4	24		**684C**	2015	11	1	
**615D**				missing	**651D**				missing	**684D**	2015	11	1	
**615E**				missing	**651E**				missing	**684E**	2015	11	1	
**622A**	2014	10	30		**661A**	2015	6	23		**693A**	2015	12	21	
**622B**	2014	10	30		**661B**	2015	6	23		**693B**	2015	12	21	
**622C**	2014	10	30		**661C**	2015	6	23		**693C**	2015	12	21	
**622D**				missing	**661D**	2015	6	23		**693D**	2015	12	21	
**622E**				missing	**661E**				missing	**693E**	2015	12	21	
**627A**	2014	12	2		**665A**	2015	7	13		**710A**	2016	4	12	
**627B**	2014	12	2		**665B**	2015	7	13		**710B**	2016	4	12	
**627C**	2014	12	2		**665C**	2015	7	13		**710C**	2016	4	12	
**627D**				missing	**665D**	2015	7	13		**710D**	2016	4	12	
**627E**				missing	**665E**	2015	7	13		**710E**	2016	4	12	

Figure S1. OTU Richness using fungal internal transcribed spacer (ITS) 96% identity OTUs analysis of environmental DNA samples from Piver's Island Coastal Observatory-Longitudinal Oceanographic Variability Experiment (PICO-LOVE) transect stations (July 2014- April 2016), with Station A closest to shore out to Station E at the continental shelf break. The box for each sampling site represents the 25th and 75th percentiles of observations; the whiskers represent the lowest and highest values. In the parenthesis, n equals the number of samples collected at each station. OTU Richness was not statistically different at any station (ANOVA, $p > 0.05$).

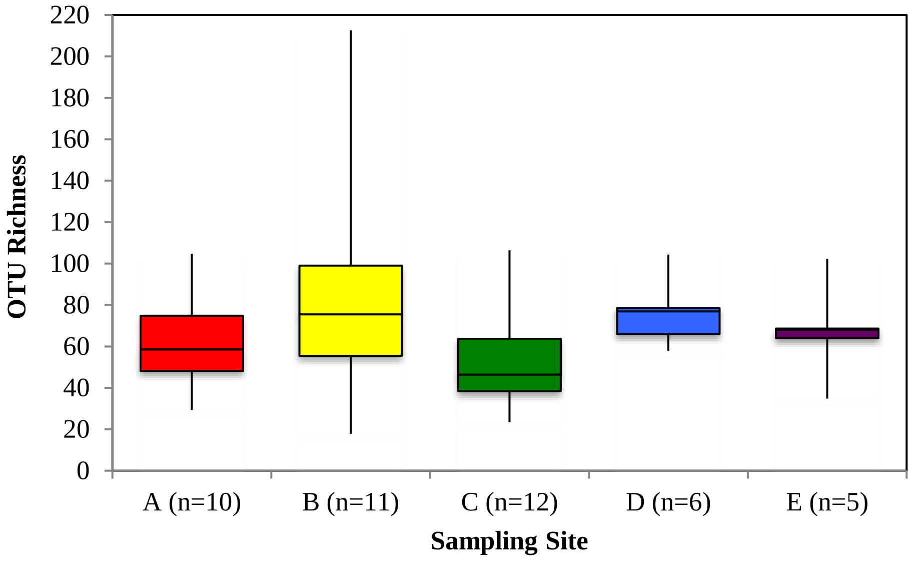

Supplement: SUPPLEMENTAL FILE 1 — Supplemental material. Download SPECTRUM01470-21_Supp_1_seq9.pdf, PDF file, 0.3 MB [file spectrum01470-21_supp_1_seq9.pdf]
